# Latitudinal Patterns in Phenotypic Plasticity and Fitness-Related Traits: Assessing the Climatic Variability Hypothesis (CVH) with an Invasive Plant Species

**DOI:** 10.1371/journal.pone.0047620

**Published:** 2012-10-22

**Authors:** Marco A. Molina-Montenegro, Daniel E. Naya

**Affiliations:** 1 Centro de Estudios Avanzados en Zonas Áridas, Facultad de Ciencias del Mar, Universidad Católica del Norte, Coquimbo, Chile; 2 Departamento de Ecología y Evolución, Facultad de Ciencias and Centro Universitario de la Regional Este, Universidad de la República, Montevideo, Uruguay; University of Sydney, Australia

## Abstract

Phenotypic plasticity has been suggested as the main mechanism for species persistence under a global change scenario, and also as one of the main mechanisms that alien species use to tolerate and invade broad geographic areas. However, contrasting with this central role of phenotypic plasticity, standard models aimed to predict the effect of climatic change on species distributions do not allow for the inclusion of differences in plastic responses among populations. In this context, the climatic variability hypothesis (CVH), which states that higher thermal variability at higher latitudes should determine an increase in phenotypic plasticity with latitude, could be considered a timely and promising hypothesis. Accordingly, in this study we evaluated, for the first time in a plant species (*Taraxacum officinale*), the prediction of the CVH. Specifically, we measured plastic responses at different environmental temperatures (5 and 20°C), in several ecophysiological and fitness-related traits for five populations distributed along a broad latitudinal gradient. Overall, phenotypic plasticity increased with latitude for all six traits analyzed, and mean trait values increased with latitude at both experimental temperatures, the change was noticeably greater at 20° than at 5°C. Our results suggest that the positive relationship found between phenotypic plasticity and geographic latitude could have very deep implications on future species persistence and invasion processes under a scenario of climate change.

## Introduction

Populations exposed to environmental changes may respond in four not mutually exclusive ways: they can become extinct, migrate to new areas, adapt via genetic change, or persist via phenotypic plasticity [1]. Although the last two alternatives avoid local extinction, current evidence suggests that for most populations coping with accelerated changing conditions, and thus local persistence, will be closely related to the amount of plasticity for fitness-related traits [2]–[4].

However, contrasting with the central role of phenotypic plasticity in population persistence, standard models aimed to predict the effect of climatic change on species distribution (i.e., the climate envelope models) do not allow for the inclusion of differences in plastic responses among populations. Obviously this is not a fanciful constraint; the quantification of plasticity for several traits in several populations is a difficult task, and thus available data in this regard are still very scarce (e.g., provenance tests). An encouraging pathway that would trade-off the large amount of data needed to include differences in plastic responses among populations (of each species to be modeled) with the fairly low predictive power of current climate envelope models [5], is the identification of global patterns in phenotypic plasticity, which could be easily incorporated into the models if they exist.

In this context, the climatic variability hypothesis (CVH) could be considered a timely and promising hypothesis, since it directly connects phenotypic plasticity with climatic and geographic variables at a global scale. Specifically, the CVH states that as the range of climatic fluctuation experienced by terrestrial animals increases with latitude, individuals at higher latitudes should have broader ranges of thermal tolerance and acclimation abilities that enable them to cope with the fluctuating environmental conditions [6; see also 7–8]. Given that tolerance ranges and acclimation responses are ultimately linked to mechanisms of morphological, physiological and/or behavioral flexibility, the central idea of the CVH has been recently extended to phenotypic plasticity in general [9]–[11]. Up to the present, empirical evidence supporting the CVH may be clustered in three major groups: (1) studies that directly evaluated the relationship between latitude and thermal tolerance range in ectothermic animals [e.g., 12–18]; (2) studies that directly evaluated the relationship between latitude and phenotypic flexibility for non-thermal traits in ecto- and endothermic animals [e.g., 19–22]; and (3) studies that analyzed different ecological patterns at the population and community levels that are expected to emerge from the CVH [e.g., 7, 23–28].

An important gap that still remains in our knowledge is the direct applicability of the CVH to plant species. On theoretical grounds there are two contrasting ideas about how plant species should change with geographic latitude to cope with the environment. On one hand, the limited vagility of plants may result in a great degree of adaptation to local conditions, resulting in a great population differentiation, which could preclude the existence of a latitudinal pattern in trait plasticity [29]–[31]. On the other hand, the limited vagility of plants may imply a reduced ability to avoid environmental influences, and thus the pattern predicted by the CVH could be more clearly observed than in animal species [10], [32]. Although some studies have been conducted to assess the relationship between plasticity and climatic heterogeneity in plant species [33]–[35], to the best of our knowledge no empirical study has evaluated the validity of CVH in these organisms.

Accordingly, the aim of the present study was to analyze how plasticity for several ecophysiological and fitness-related traits changes with latitude in the invasive *Taraxacum officinale* (dandelion complex). Specifically, we evaluated plasticity –due to differences in environmental temperature (5 and 20°C)– in photosynthetic rate, water use efficiency, foliar angles, plant biomass, number of flowers and seed output in five populations that occur along a latitudinal gradient (from 0° to 54°S). We predicted that the reduction in the duration of the growing season observed at higher latitudes should determine that populations at higher latitudes will be more efficient at exploiting favorable thermal conditions (20°C) than populations from lower latitudes. Additionally, we predicted that higher thermal heterogeneity at higher latitudes should determine that populations at higher latitudes would be more plastic. Thus we expect to observe, in agreement with the CVH, a positive relationship between phenotypic plasticity and geographic latitude.

## Methods

### Populations, Traits and Experimental Environments

**Figure 1:**
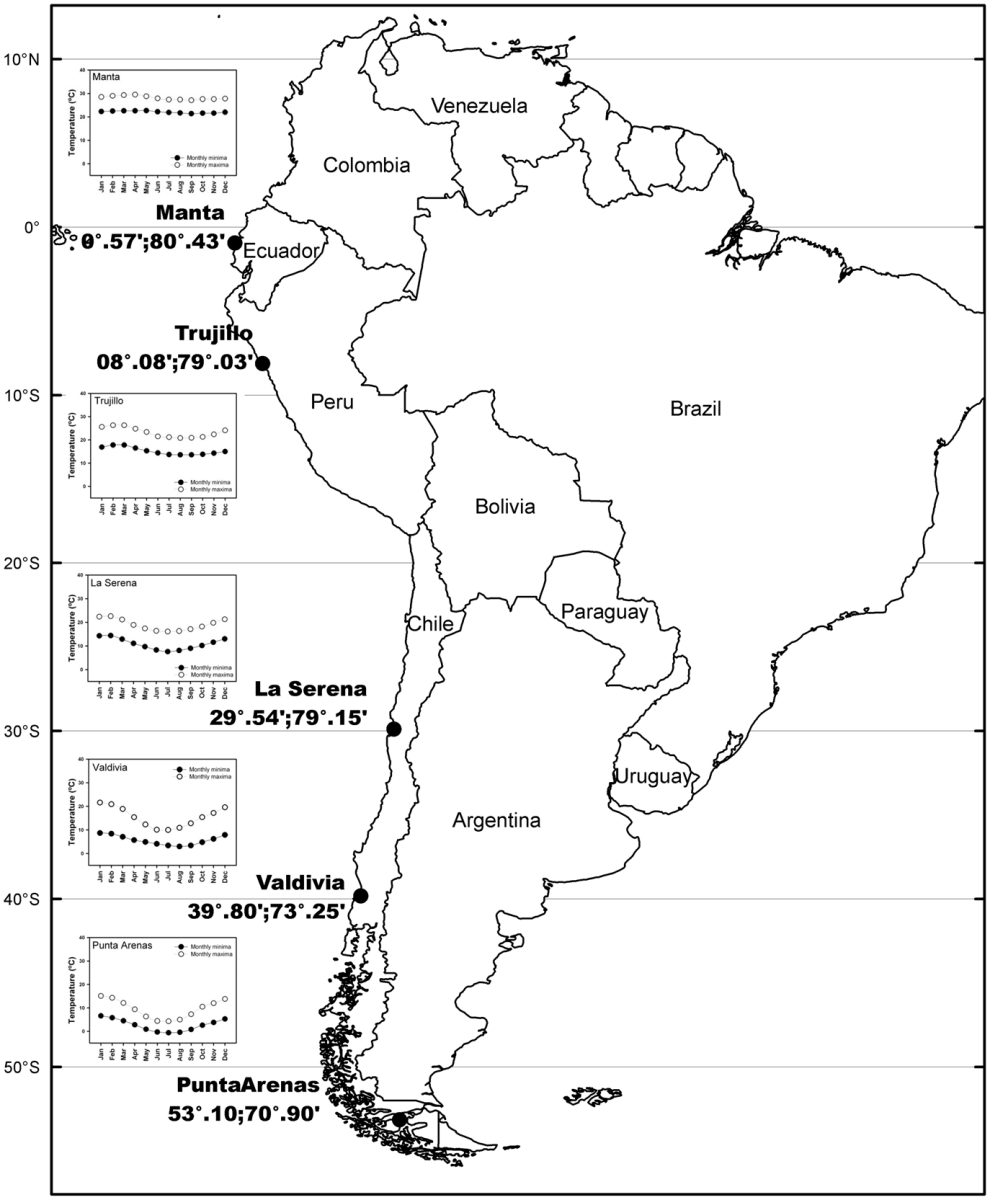
Map showing the five sampled localities along the latitudinal gradient. Monthly minimum and maximum temperatures are given for each locality.

*Taraxacum officinale* is a member of the Asteraceae originally from Europe that has spread worldwide. It is one of the top invasive species around the world [36]. This plant can be found growing in sites with changing climatic characteristics, disturbance regimes, and along a wide range of altitudes and latitudes. Seeds of *T. officinale* were collected in five localities: Manta (Ecuador), Trujillo (Perú), La Serena (Chile), Valdivia (Chile) and Punta Arenas (Chile). The hemispheric latitudinal gradient covers from *ca.* 0° to *ca.* 54° S, including a notorious and significant thermal gradient (Fig. 1). All seeds were collected at sea level to reduce altitudinal effects (Fig. 1). Preliminarily, cytogenetic analysis showed that individuals from all localities sampled in this study have the same ploidy level (n = 24 chromosomes) and size of chromosomes, suggesting that all individuals sampled belong to the same species (data not shown).

A small number of seeds (four to five) per individual plant collected from a relatively large number of sampled plants (50–55) per population provided the initial pool of seeds. As *T. officinale* has apomictic reproduction [37], samples were taken from widely separated plants to avoid sampling the same genet twice. Seeds in each locality were collected from 3 populations separated by 1 km each. Finally, all seeds collected in the 3 populations of each locality were pooled and randomized before sorting them into experimental treatments. This was done because the aim of this study was to compare responses in a number of genotypes from different localities growing along the latitudinal gradient rather than to isolate genotypic effects from phenotypic effects [33], [38]. No specific permits were required for seed collection in the localities sampled in this study and confirm that all populations are not privately-owned or protected in any way. Additionally, we confirm that the field studies did not involve endangered or protected species.

Seeds from all localities were germinated at 24±2°C on wet filter paper in Petri dishes and planted in 300-mL plastic pots filled with potting soil. First generation plants (F1) were generated from this initial seed pool and were grown in a greenhouse at the Universidad de Concepción (Concepción, Chile) under controlled conditions of light and temperature (1320 µmol m-2s-1±55 and 22±2°C, respectively). These plants were again put in 300-mL plastic pots filled with potting organic soil and irrigated every two days with 50 ml of water. After five months these plants produced the achenes that were used to obtain experimental plants (F_2_). One week after of appearance of the first true leaf, F_2_ seedlings were transferred to growth chambers (Forma Scientific Inc.) with a photon flux density (PFD) of 170 µmol m-2 s-1 and 16/8 h light/dark photoperiod. The temperature treatment consisted of transferring 20 individuals from each locality described above to a growth chamber set at 5 or 20°C for 90 days. These temperatures were chosen because they are close to the mean temperatures in each extreme of gradient. Plants were irrigated daily and supplemented with Phostrogen® (Solaris, NPK, 14∶10∶27) using 0.2 g L^−1^ once every 15 days. Plastic pot positions were randomized within the experimental plot every four days. Interplot distances were sufficient to prevent mutual shading. After 90 days we recorded three ecophysiological traits, net photosynthesis, water use efficiency (photosynthesis/foliar transpiration) and foliar angles, and three fitness-related traits (total dry biomass, flower production and seed output).

### Climatic Data

For each sampled population we downloaded data from the WorldClim data base (http://www.worldclim.org/) on the following climatic variables: annual mean temperature (Tmed, in °C), minimum temperature of the coldest month (Tmin, in °C), maximum temperature of the warmest month (Tmax, in °C), temperature seasonality (TS: standard deviation of the mean monthly temperature, in °C), temperature annual range (TAR: difference between maximum temperature of warmest month and minimum temperature of the coldest month, in °C), accumulated annual precipitation (Rainfall, in mm), and rainfall seasonality (RS: standard deviation of the mean monthly rainfall, in mm) (Table 1).

**Table 1:** Climatic variables for each locality (see Methods for abbreviations).

	Manta	Trujillo	La Serena	Valdivia	Punta Arenas
**Tmed (**°**C)**	25.1	19.2	14.9	10.5	6.1
**Tmin (**°**C)**	21.4	13.6	7.6	3.0	−0.6
**Tmax (**°**C)**	29.5	26.3	22.6	21.6	15.1
**TS (**°**C)**	62.7	187.2	241.3	309.3	327.1
**TAR (**°**C)**	8.1	12.7	15.0	18.6	15.7
**Rainfall (mm)**	1788	6	83	2211	431
**RS (mm)**	64.0	180.9	119.5	61.3	15.6

### Plasticity Estimations and Statistical Analyses

Phenotypic plasticity was considered as the environmentally-induced change in the expression of phenotypic traits at the end of the experimental period, whatever the mechanistic causes (e.g., ontogenetic, allometric) behind this differential expression [39]. Phenotypic plasticity was estimated for each trait and locality as the percentage of change in mean trait value from one environment to the other; that is, P = [(X_20_– X_5_)/X_20_] * 100, where P is plasticity, X_20_ is the mean trait value at 20°C and X_5_ is the mean trait value at 5°C [40]. In addition, a measurement of overall plasticity was estimated for each population as the arithmetic average of the percentage of change observed for all the traits. A bootstrapping procedure, with 1000 iterations was used in order to obtain error estimations of plasticity for each trait and population (i.e., a percentage of change was calculated from two randomly selected individuals, one in each temperature treatment, in each iteration). The error estimation for overall plasticity was calculated as the arithmetic mean of the errors obtained for all the traits. The relationships between mean trait values at each temperature and trait plasticity with geographic latitude and climatic variables were evaluated separately using the Pearson product moment coefficient.

## Results

**Figure 2:**
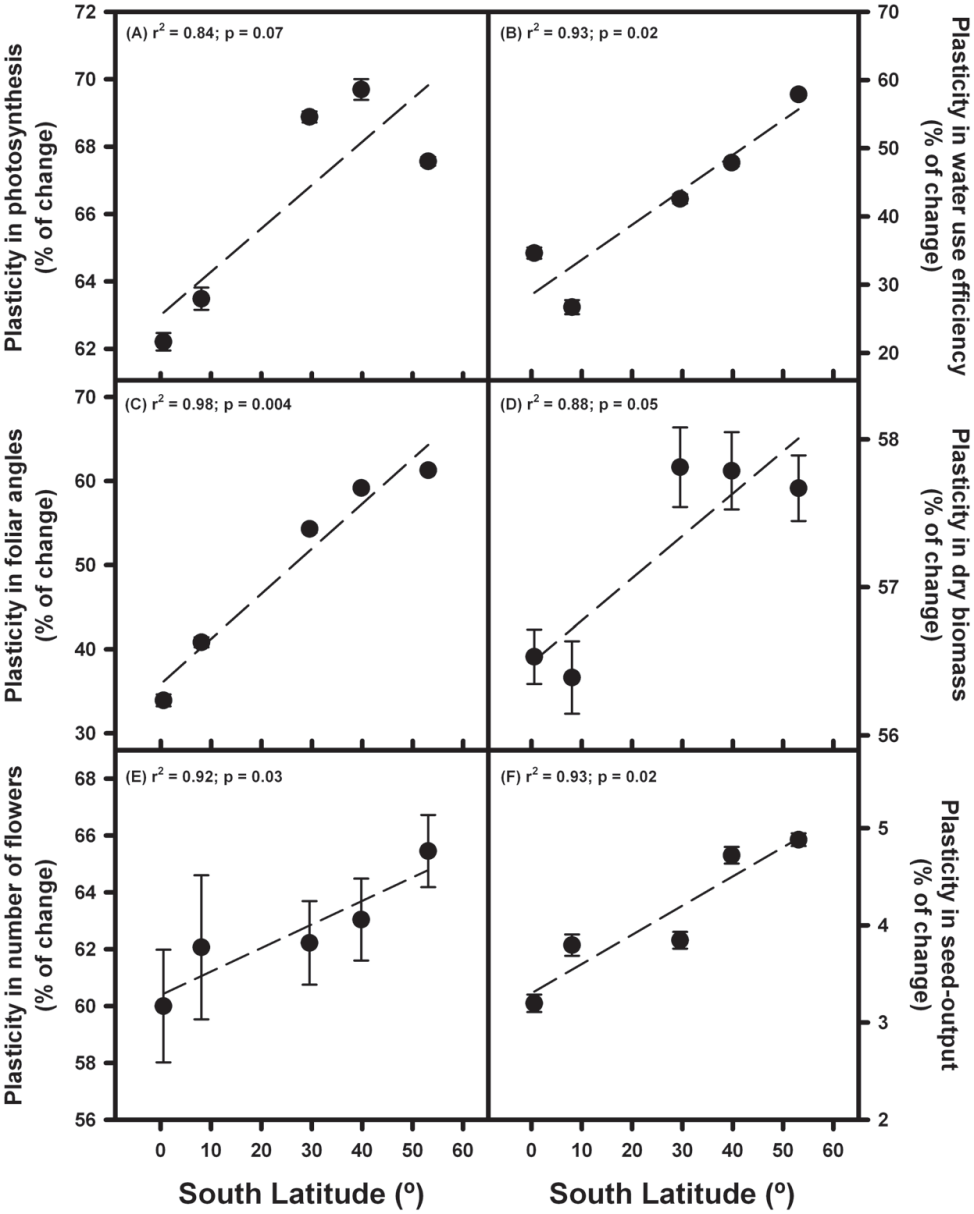
Relationship between trait plasticity (measured as percentage of change) and latitude: (A) photosynthetic efficiency, (B) water use efficiency, (C) foliar angle, (D) plant biomass, (E) number of flowers, (F) seed output.

Except for water use efficiency and foliar angle at 5°C, for which no latitudinal changes were observed, mean trait values increased with latitude at both experimental temperatures (Table 2). In addition, given that these increases in mean values were noticeably greater at 20°C than at 5°C, phenotypic plasticity also increased with latitude for all the six analyzed traits (Fig. 2). Regarding the relationship between phenotypic plasticity and climatic variables, it was observed that plasticity in photosynthetic rate was positively related to temperature annual range, plasticity in water use efficiency was negatively correlated with maximum temperature of the warmest month, and plasticity in foliar angle, flower production, seed output and overall plasticity were positively related to thermal seasonality and negatively related to environmental temperatures (Table 3).

**Figure 3:**
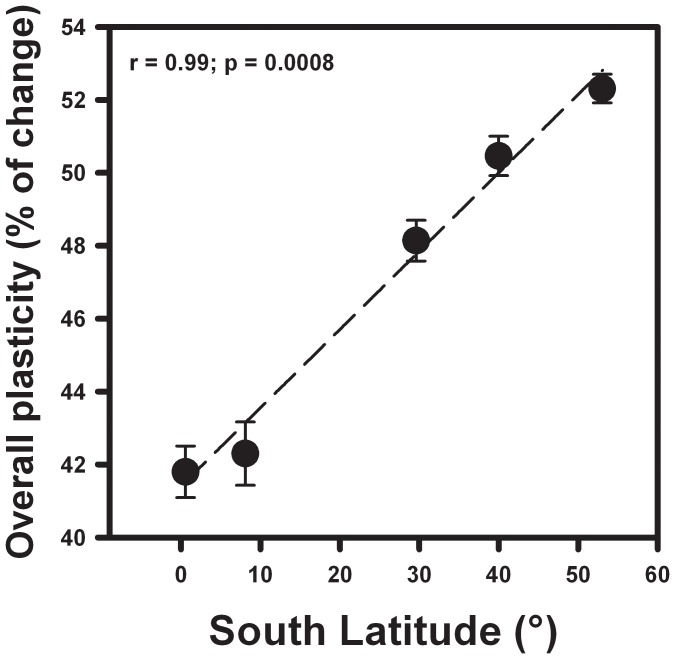
Relationship between overall plasticity (measured as the arithmetic mean of the percentage of change for all the six traits) and latitude.

Neither annual rainfall nor rainfall seasonality were correlated with plasticity for any trait (Table 3). In any case, the number of significant correlations between any single climatic variable and trait plasticity was always less than those observed for latitude, and moreover, latitude was by far a better predictor of overall plasticity than any climatic variable (Table 3, Fig. 3).

**Table 2:** Mean trait values measured at 5°C and 20°C for each locality, and the Pearson product moment correlation coefficient (together with the associated probability and the slope of the regression) for the correlation between mean trait values and latitude.

	Manta	Trujillo	La Serena	Valdivia	Pta. Arenas	r	P-value	Slope
**A max (µmol m^−2^ s^−1^) at 5°C**	3.03 (0.06)	2.96 (0.08)	4.03 (0.08)	5.18 (0.19)	5.38 (0.06)	0.90	<0.000001	0.05
**A max (µmol m^−2^ s^−1^) at 20°C**	8.03 (0.14)	8.11 (0.20)	12.94 (0.13)	17.09 (0.25)	16.59 (0.20)	0.94	<0.000001	0.19
**WUE (µmol CO_2_/mmol H_2_O) at 5°C**	1.22 (0.05)	1.28 (0.06)	1.19 (0.05)	1.23 (0.05)	1.23 (0.06)	0.04	0.71	0.0005
**WUE (µmol CO_2_/mmol H_2_O) at 20°C**	1.87 (0.05)	1.75 (0.05)	2.07 (0.04)	2.37 (0.04)	2.91 (0.04)	0.86	<0.000001	0.02
**Foliar angle (°) at 5°C**	12.33 (0.44)	11.20 (0.38)	12.07 (0.40)	11.27 (0.34)	11.67 (0.35)	−0.08	0.47	−0.007
**Foliar angle (°) at 20°C**	18.67 (0.42)	18.93 (0.43)	26.40 (0.55)	27.60 (0.54)	30.13 (0.46)	0.92	<0.000001	0.24
**Plant biomass (mg) at 5°C**	37.93 (0.55)	38.67 (0.81)	42.67 (0.91)	45.53 (0.92)	52.07 (0.92)	0.83	<0.000001	0.26
**Plant biomass (mg) at 20°C**	87.27 (0.78)	88.67 (0.61)	101.13 (1.47)	107.87 (1.46)	123.00 (1.36)	0.93	<0.000001	0.67
**Number of flowers at 5°C**	0.80 (0.14)	0.73 (0.18)	1.13 (0.17)	1.13 (0.17)	1.27 (0.18)	0.30	0.01	0.01
**Number of flowers at 20°C**	2.00 (0.17)	1.93 (0.18)	3.00 (0.17)	3.07 (0.18)	3.67 (0.16)	0.71	<0.000001	0.03
**Seed-output at 5°C**	0.901 (0.001)	0.901 (0.003)	0.915 (0.002)	0.922 (0.003)	0.926 (0.002)	0.80	<0.000001	0.0005
**Seed-output at 20°C**	0.931 (0.003)	0.937 (0.003)	0.952 (0.002)	0.968 (0.002)	0.973 (0.002)	0.86	<0.000001	0.001

A max = photosynthetic rate; WEU = water use efficiency.

**Table 3:** Pearson product moment correlation coefficient (and associated probability) for the correlations between ecophysiological and fitness-related trait plasticity (measured as percentage of change) and climatic variables.

	A max	WUE	Foliar angle	Plant biomass	Number of flowers	Seed output	Overall plasticity
**Tmed (°C)**	−0.81 (0.10)	−0.86 (0.06)	−0.97 (0.007)	−0.81 (0.10)	−0.96 (0.01)	−0.97 (0.007)	−0.96 (0.01)
**Tmin (°C)**	−0.86 (0.06)	−0.84 (0.08)	−0.98 (0.003)	−0.84 (0.08)	−0.93 (0.02)	−0.96 (0.01)	−0.96 (0.01)
**Tmax (°C)**	−0.71 (0.18)	−0.89 (0.04)	−0.92 (0.03)	−0.76 (0.13)	−0.98 (0.005)	−0.91 (0.03)	−0.94 (0.02)
**TS (°C)**	0.86 (0.06)	0.76 (0.13)	0.97 (0.006)	0.81 (0.10)	0.91 (0.03)	0.96 (0.01)	0.92 (0.03)
**TAR (°C)**	0.92 (0.03)	0.63 (0.26)	0.92 (0.03)	0. 81 (0.09)	0.73 (0.16)	0.88 (0.05)	0.84 (0.08)
**Rainfall (mm)**	0.05 (0.94)	0.14 (0.82)	−0.06 (0.93)	0.06 (0.92)	−0.28 (0.64)	0.04 (0.94)	0.02 (0.97)
**RS (mm)**	−0.31 (0.61)	−0.83 (0.08)	−0.46 (0.44)	−0.49 (0.40)	−0.43 (0.47)	−0.49 (0.40)	−0.62 (0.26)

A max = photosynthetic rate (µmol m^−2^ s^−1^), WUE = water use efficiency (µmol CO_2_/mmol H_2_O).

## Discussion

The Earth is undergoing dramatic environmental changes (referred to as global change) which are mainly related to five different (but interacting) phenomena: climate change, land use change, resource overexploitation, pollution, and invasive species [41], [42]. Two results obtained in the present study are relevant in this scenario of global change. First, in agreement with the climatic variability hypothesis (CVH), a clear association was found between latitude and plasticity for all the ecophysiological and fitness-related traits analyzed. Second, all the sampled populations of the highly invasive species considered here showed a great degree of plasticity for all the traits evaluated, regardless of their specific location along the latitudinal gradient. Thus, in what follows we discuss the implications of these results within the contexts of the CVH and species invasiveness ability.

### Latitudinal Patterns in Phenotypic Plasticity

As mentioned above, the present study demonstrates for the first time in a plant species the occurrence of the latitudinal pattern of phenotypic plasticity predicted by the CVH. Moreover, correlation coefficients (ranging between 0.84 and 0.99) support the idea that latitudinal patterns in plasticity maybe stronger in plants than in animal species [10]. The positive relationship between plasticity and latitude was due to a slight increase (or no change) in trait values with latitude at 5°C but a strong increase at 20°C. This result is logical from a biological point of view, since: (1) Environmental temperatures of 5°C are close to monthly mean temperatures in high latitude localities such as Punta Arenas and Valdivia, but are far from monthly temperatures (even from monthly minima) in the other localities (Figure 1). Thus a selective advantage associated with an increase in trait values at this temperature should only be expected in the former localities. In line with this idea, it should be noted that the only traits that did not change with latitude were foliar angle and water use efficiency, i.e., traits for which a rise in mean values with latitude probably do not represent a selective advantage. (2) Environmental temperatures of 20°C are higher than monthly maxima in Punta Arenas, close to monthly maxima during four months in Valdivia and during seven months in La Serena, close to the monthly mean in Trujillo, and close to the monthly minima in Manta (Figure 1). Thus, a temperature of 20°C is probably associated with the short favourable season at middle and high latitudes, but only with normal or even unfavourable conditions at lower latitudes. Consequently, positive selection for higher trait values at this temperature is expected to be very strong in high latitude localities, but only weak (or null) in low latitude localities. It has been proposed that populations growing along latitudinal gradients in its poleward limit of distributions should show a trade-off between cold resistance and growth, thus any amelioration in thermal stress conditions will be positive for these populations, improving their ecophysiological performance compared with other populations distributed at lower latitudes (see [43]. Unfortunately, our experimental design did not contemplate environmental temperatures greater than 20°C, for which a strong positive selection for higher traits values may be expected for all the localities. Thus, it is possible that the inclusion of a higher temperature (e.g., 30°C) in the experimental setup could attenuate the strong latitudinal pattern for plasticity reported here.

On the other hand, reported data suggest that latitude is a better predictor of phenotypic plasticity than climatic variables. This result, usually reported in macrophysiological studies [e.g., 19, 44, 45], appears to be related to at least three different facts [21]. First, latitude is probably a better predictor of long-term regimes of climatic variables than current climate values provided by weather stations. Second, latitude is correlated with several other climatic, ecological, and historical factors that could affect phenotypic plasticity. Third, the smooth variation of climatic variables in space suggests that latitude may represent a weighted variable of climatic conditions acting over spatial scales more similar to those at which adaptation is expected to occur. In any case, recent studies evaluating the effect of latitude and climatic variability on phenotypic plasticity for areas where both sets of variables are not directly correlated have found that climatic variables, and not latitude, were the best predictors of phenotypic plasticity [20], [23]. Thus, as stated by the CVH, climatic variability appears to be the proximal cause behind the latitudinal patterns in phenotypic plasticity. Thus, phenotypic plasticity can play an important role both in invasive and native plant species along gradients [42], [46]. For example, Santamaría et al. (2003) [32] showed the positive role of phenotypic plasticity in aquatic plants across a latitudinal gradient. Additionally, Mou et al. 2012 [47], demonstrated that plasticity in morphological and physiological traits would improve the performance and resource acquisition in environmental variability conditions.

Finally, the results found in this study are in line with recent papers that also report an increase in phenotypic plasticity with latitude for different traits and taxonomic groups (see Introduction). Obviously, this does not mean that all the existing evidence supports the prediction of the CVH. For instance, studies analyzing acclimation abilities in ectothermic animals do not show a clear pattern of latitudinal variation [12], [48]–[50]. Although it is true that these studies had a more reduced taxonomic scope (e.g., one genus or one family) and geographic extension (e.g., one region or one continent) than most of the studies that support the CVH –suggesting that differences in results may be due in part to difference in the temporal and spatial scales considered– it could be expected that the prediction of the CVH does not hold for all phenotypic characters. As for phenotypic plasticity itself –although the lack of plasticity for one or more traits does not deny the adaptive value of plasticity in general– the lack of latitudinal patterns in plasticity for some traits does not deny the validity of the CVH in general terms. We believe that the evidence supporting the CVH is enough to warrant that it be seriously considered in models aimed at predicting the effect of global change (and particularly the effect of global warming) in future species distributions. A simple way to do this is to modify the current climate envelope models by increasing the potential niche of each population in parallel to latitude, but the specific details on how this could be done this exceed the aim of the present study.

### The Relationship between Phenotypic Plasticity and Invasiveness

Although it has been reported that *T. officinale* may reduce the fitness of native herbs in many ways [35], [50], little is known about the mechanisms associated with its wide distribution (but see [51].) Based on our results we suggest that *T. officinale* is capable of invading a broad latitudinal gradient mainly because, being an r-selected species, this species is also able to exhibit a great level of phenotypic plasticity. In fact, in a recent paper Richards et al. (2006) [52] suggested that biological invasions would be driven by phenotypic plasticity, playing an important role in successful plant invasions in wide clines. Thus plasticity in functional traits may enhance ecological niche breadth and therefore confer a fitness advantage [52]. Geographic gradients in abiotic conditions across a wide range could impose divergent selection pressures and promote genetically based differentiation among introduced populations. A classic manifestation of this is the evolution of geographic clines, often found in native species occurring across altitudinal gradients [53], [54]. However, whether introduced plant populations rapidly evolve clines in response to environmental conditions across their introduced range has seldom been studied [54], [55]. Furthermore, whether clines in traits among introduced plant populations broadly converge on those expressed among native conspecifics occurring over similar latitudinal or altitudinal gradients is unknown. Nevertheless, this may be the case for *T. officinale*, which shows not only high plasticity, but also local adaptation along a broad latitudinal gradient. Phenotypic plasticity and ecotypic differentiation are two complementary strategies to face environmental heterogeneity [29], [55]–[57]. It has been shown that plasticity can initially allow exotic species to become naturalized across the non-native range [57] and, once naturalized, genetic recombination of heritable phenotypes may respond to local selection pressures giving rise to ecotypes with higher fitness [58].

The triploid condition of *T. officinale* individuals from all localities studied suggests they are apomictics, which usually is associated with low genetic variability and high plasticity as a successful strategy to cope with changing environments [35]. Nevertheless, unexpectedly high levels of genetic variation have been found in other apomictic species generated by subsexual reproduction [59], including genetic segregation and hybridization between sexual and apomictic individuals [60], [61]. Exceptions to the general pattern of high cytogenetic variability in widely distributed alien species do exist. They tend to occur in species with very high levels of phenotypic plasticity (e.g., *Taraxacum*) which have a low level of cytogenetic variability [62].

The high invasive capacity of Eurasian species is considered to be a result of an evolutionary history on a large continental mass that suffered major upheavals during the glacial period and longer association between humans and plants than in the New World [63]; consequently, aliens of Eurasian origin, because of their higher competitiveness, vagility and plasticity are likely to invade areas even when interchange is fairly limited [64]. Finally, the fact that species that have been introduced into South America and can adapt to the wide range of environmental conditions found along fifty-five degrees of latitude could be surprising. Surely the high plasticity in the ecophysiological and fitness-related traits of *T. officinale* individuals from different populations suggests that some invaders could definitely adapt to changing environmental conditions found along broad gradients worldwide.

### Conclusions

The present study shows that the prediction of the climatic variability hypothesis appears to hold for plant species, finding a strong positive relationship between phenotypic plasticity and geographic latitude. As discussed, this result could have very profound implications on future species persistence under a scenario of climate change. In addition, obtained data support the idea that the great invasiveness ability reported for *T. officinale* along broad gradients could be related with both, to being a weedy r-selected species and to having high plasticity levels for several ecophysiological and fitness-related traits.
